# Splenic Infarction: A Rare Complication of Infectious Mononucleosis

**DOI:** 10.7759/cureus.37414

**Published:** 2023-04-11

**Authors:** Marta Batista, Cristina Silva, Filipa Madalena F Gonçalves, Marcia Machado, Sara Freitas, Juliana Silva, Jorge Cotter

**Affiliations:** 1 Internal Medicine, Hospital Senhora da Oliveira Guimarães, Guimarães, PRT; 2 Internal Medicine, Hospital Senhora da Oliveira Guimaraes, Guimaraes, PRT

**Keywords:** acute airway obstruction, epstein-barr virus, tonsillar abscess, infectious mononucleosis, splenic infarction

## Abstract

Infectious mononucleosis (IM) is caused by Epstein-Barr virus (EBV), and the condition is characterized by sore throat, fever, lymphadenopathy, and atypical lymphocytosis. These infections are common in early childhood, with a second peak occurring in late adolescence. EBV is spread by contact with oral secretions. Most cases of IM are self-limited. However, there are associated complications, some of which can be serious and fatal. We report the case of a 20-year-old man with splenic infarction and exuberant peritonsillar abscess secondary to an EBV infection. This case highlights the importance of accurate diagnoses and frequent monitoring in IM patients, given the risk of airway obstruction.

## Introduction

Infectious mononucleosis (IM) is caused by Epstein-Barr Virus (EBV) and is transmitted by salivary secretions. EBV infects the epithelium of the oropharynx and salivary glands and then spreads through the bloodstream [1]. Lymphoid tissue hyperplasia occurs due to the proliferation and expansion of EBV-infected B cells with reactive T cells [1].

In infants and young children, EBV infections are mostly asymptomatic. However, most EBV infections in young adults (75%) present as IM [1]. Clinical manifestations include odynophagia, asthenia, headache, generalized myalgia, abdominal pain, morbilliform or papular rash, nausea, or vomiting. These manifestations can accompany cervical adenopathy, fever, pharyngitis or tonsillitis, splenomegaly and/or hepatomegaly [1-5]. Most patients have symptoms for two to four weeks, and most cases are benign and self-limited [1,5]. However, there may be several potentially fatal complications, such as meningitis, encephalitis, splenic infarction, splenic rupture, airway obstruction, bacterial superinfection, hepatitis, and myocarditis [1]. Splenic infarction occurs when the splenic artery or its branches become occluded by an embolus or in situ thrombosis [4]. Here, we report the case of a 20-year-old man with splenic infarction and exuberant peritonsillar abscess secondary to an EBV infection.

## Case presentation

A 20-year-old man presented to the emergency department with fever (up to 39.0 ºC), holocranial headaches with phonophobia and photophobia, and generalized myalgia that had started four days prior to presentation. He had acne, which was treated with isotretinoin 25 mg once daily. He denied smoking, and alcohol and drugs consumption. He had no surgical history or previous hospitalizations and no drug allergies. He denied risky sexual contacts, recent travels and contacts with animals.

The patient was hemodynamically stable, conscious, and oriented with no neurological focus on examination. Physical examination results were unremarkable, with no hypertrophy or tonsil exudates and no palpable cervical lymphadenopathy or rash.

His laboratory test results are presented in Table 1. His atypical lymphocytes, C-reactive protein, aspartate aminotransferase, alanine aminotransferase, lactic dehydrogenase, and alkaline phosphatase levels were elevated. The patient did not have respiratory failure, acid-base, or electrolyte disturbances. He underwent cerebral and contrast-enhanced abdominopelvic computed tomography (CT) that was unremarkable. A lumbar puncture in the lateral decubitus position to analyze his cerebral spinal fluid revealed no pathological findings. Paul-Bunnell reaction was positive, and peripheral blood smears showed atypical lymphocytes with slightly basophilic cytoplasm, lax nuclear chromatin, and mild platelet anisocytosis with rare platelet aggregates. His EBV titers specific for IgM antibodies were elevated, his IgG titers were negative and IgG antibodies to viral capsid antigen (VCA) were positive.

**Table 1:** Laboratory investigations INR, international normalized ratio; NM, not measured

Analyte	Patient results			Reference range
	On presentation	Day 3	Discharge	
Hemoglobin (g/dL)	15.8	15.2	15.8	14-18
Leukocytes (x10^3^/µL)	6.7	16.6	6.6	4.8-10.8
Neutrophils (%)	31%	31%	44%	38-70
Lymphocytes (%)	48%	38%	42%	20-40
Atypical lymphocytes (%)	20%	21%	NM	
Monocytes (%)	1%	10%	8%	2-6
Platelets (x10^3^/µL)	151	206	185	150-300
C-reactive protein (mg/L)	17.6	24.2	0.5	<3
Total bilirubin (mg/dL)	1.12	0.84	0.50	0.3-1.2
Aspartate aminotransferase (IU/L)	198	111	22	12-40
Alanine aminotransferase (IU/L)	319	522	43	7-40
Gamma-glutamyl transferase (IU/L)	314	400	60	0-73
Lactic dehydrogenase (IU/L)	431	425	205	120-246
Alkaline phosphatase (IU/L)	242	346	64	46-116
Albumin (g/dL)	4.6	4.6	4.7	3.4-5
INR	1.4	1.3	1.1	

**Figure 1:**
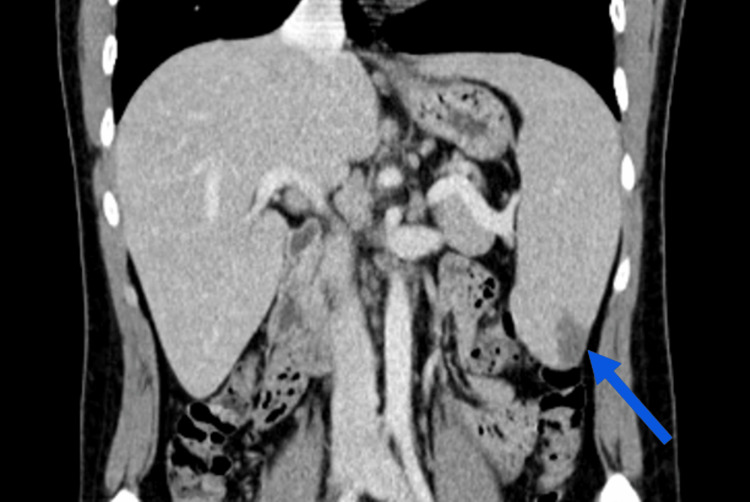
Coronal contrast-enhanced chest and abdominopelvic computed tomography showing a hypodense area on the posteroinferior aspect of the spleen (splenic infarction; blue arrow)

On the third day of hospitalization, patient's clinical condition worsened. He developed odynophagia, fever, erythematous oropharynx, tonsillar hypertrophy with bilateral purulent exudate, and left upper abdominal pain. Further laboratory workup revealed a liver dysfunction; his aspartate aminotransferase level was 111 IU/L, alanine aminotransferase was 522 IU/L, gamma-glutamyl transferase was 400 IU/L, alkaline phosphatase was 346 IU/L, and his lactic dehydrogenase level was 425 IU/L. A second contrast-enhanced chest abdominopelvic CT scan revealed splenomegaly, measuring approximately 17.5 cm in its longitudinal axis, containing a hypodense area in its posteroinferior aspect suggestive of a small splenic infarction not present in the previous CT scan (Figure 1).

**Figure 2:**
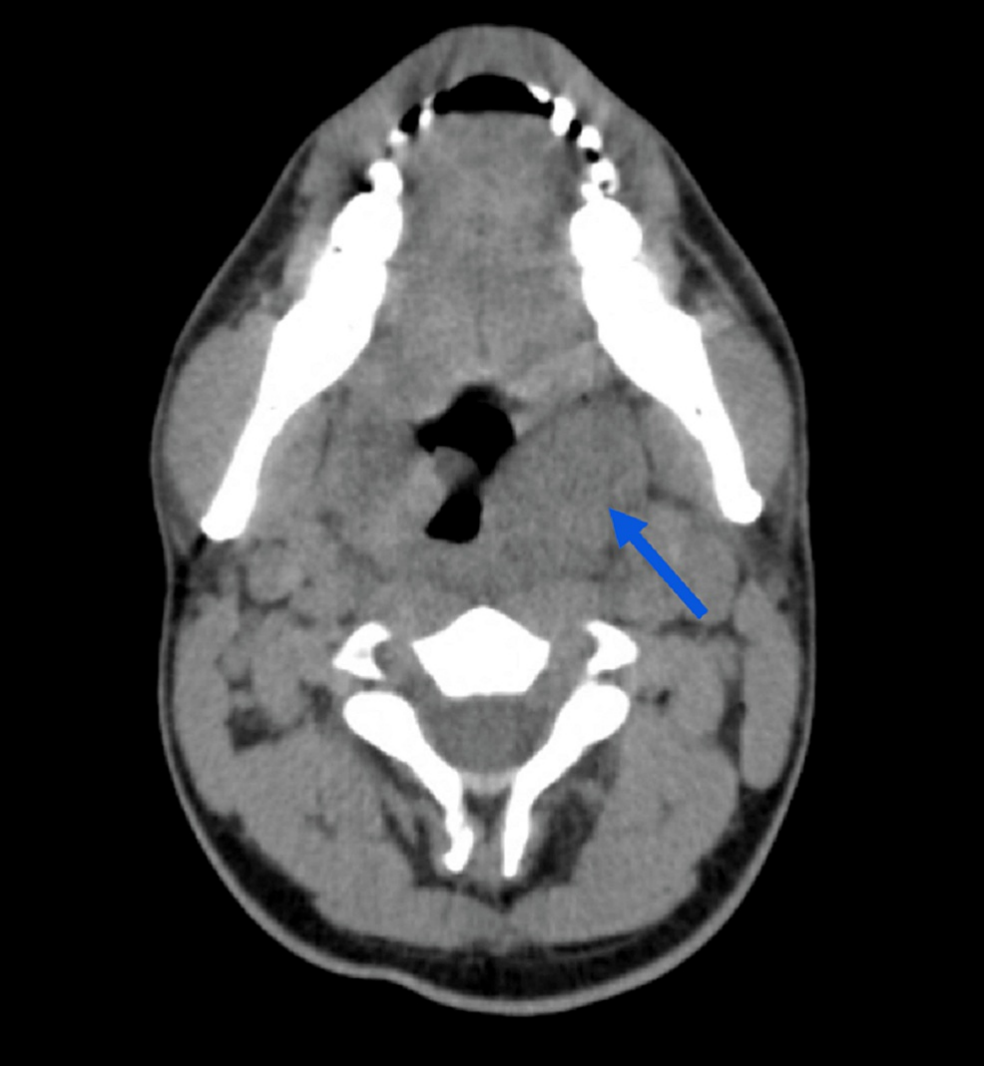
Axial computed tomography angiogram of the neck vessels showing heterogeneous and asymmetric hypertrophy of the left tonsillar pillar (blue arrow)

Transesophageal echocardiography showed no evidence of masses or thrombi, vegetations, abscesses, or pseudoaneurysms. A CT angiogram (CTA) of the neck vessels was performed to exclude thrombosis of the internal jugular vein. The CTA revealed significant left tonsillitis with tonsil and posterior peritonsillar abscess, retropharyngeal edema/exudation, and reactive lymphadenopathy with no venous thrombosis (Figure 2).

Empirical antibiotic therapy was started with metronidazole and ceftriaxone. He showed a favorable clinical improvement and his laboratory results were unremarkable. The patient was discharged for home health care to complete four weeks of antibiotic therapy.

## Discussion

Our case report describes a young man with splenic infarction and exuberant peritonsillar abscess secondary to IM. Splenic infarction is a rare complication of IM in which the splenic artery or its branches become occluded by an embolus or in situ thrombosis. Although several mechanisms have been proposed, the pathogenesis is not entirely clear [4]. One proposed mechanism is insufficient blood flow due to the increased needs caused by the hypercellular state of the spleen. Another theory is that splenic infarct occurs due to the transient state of hypercoagulability from increased antiphospholipid antibodies, lupus anticoagulants, and Factor VII. The high level of circulating immune complexes, which promotes the aggregation and adhesion of leukocytes, has been linked to splenic infarction [2-8]. Splenic infarction can occur in other clinical conditions that must be ruled out and evaluated according to each case, such as infectious endocarditis and cytomegalovirus infection, conditions that mimic EBV infection that were ruled out in our case [4].

Another complication for our patient was a peritonsillar abscess that required vigilance due to the risk of airway obstruction. In such cases, glucocorticoid therapy is contraindicated because it may predispose the patient to bacterial superinfection [1].

## Conclusions

The case reported here is of a man with rare splenic infarction and an exuberant peritonsillar abscess secondary to an EBV infection. Splenic infarction was treated conservatively because there were no complications such as rupture or hemorrhage. Overall, this case shows that an effective diagnostic approach leading to accurate diagnoses and frequent monitoring in IM patients is highly important to detect, monitor, and treat possible fatal complications.
